# The Total and Active Bacterial Community of the Chlorolichen *Cetraria islandica* and Its Response to Long-Term Warming in Sub-Arctic Tundra

**DOI:** 10.3389/fmicb.2020.540404

**Published:** 2020-12-18

**Authors:** Ingeborg J. Klarenberg, Christoph Keuschnig, Denis Warshan, Ingibjörg Svala Jónsdóttir, Oddur Vilhelmsson

**Affiliations:** ^1^Natural Resource Sciences, University of Akureyri, Akureyri, Iceland; ^2^Faculty of Life and Environmental Sciences, University of Iceland, Reykjavík, Iceland; ^3^Environmental Microbial Genomics, Laboratoire Ampère, CNRS, École Centrale de Lyon, Écully, France; ^4^BioMedical Center, University of Iceland, Reykjavík, Iceland; ^5^School of Biological Sciences, University of Reading, Reading, United Kingdom

**Keywords:** lichen, lichen microbiome, tundra, climate change, host–microbiome, lichen-associated bacteria, long-term warming

## Abstract

Lichens are traditionally defined as a symbiosis between a fungus and a green alga and or a cyanobacterium. This idea has been challenged by the discovery of bacterial communities inhabiting the lichen thalli. These bacteria are thought to contribute to the survival of lichens under extreme and changing environmental conditions. How these changing environmental conditions affect the lichen-associated bacterial community composition remains unclear. We describe the total (rDNA-based) and potentially metabolically active (rRNA-based) bacterial community of the lichen *Cetaria islandica* and its response to long-term warming using a 20-year warming experiment in an Icelandic sub-Arctic tundra. 16S rRNA and rDNA amplicon sequencing showed that the orders Acetobacterales (of the class Alphaproteobacteria) and Acidobacteriales (of the phylum Acidobacteria) dominated the bacterial community. Numerous amplicon sequence variants (ASVs) could only be detected in the potentially active community but not in the total community. Long-term warming led to increases in relative abundance of bacterial taxa on class, order and ASV level. Warming altered the relative abundance of ASVs of the most common bacterial genera, such as *Granulicella* and *Endobacter*. The potentially metabolically active bacterial community was also more responsive to warming than the total community. Our results suggest that the bacterial community of the lichen *C. islandica* is dominated by acidophilic taxa and harbors disproportionally active rare taxa. We also show for the first time that climate warming can lead to shifts in lichen-associated bacterial community composition.

## Introduction

The notion that lichens harbor diverse bacterial and fungal communities has challenged the traditional view of the lichen as a symbiosis between a fungus (mycobiont) and an alga and/or a cyanobacterium (photobiont) (González et al., 2005; Spribille et al., 2016). Nonetheless, the first lichen-associated bacteria were already discovered in the 1920s (Uphof, 1925). To date, bacterial communities of a wide-range of lichen species have been revealed by molecular approaches (Cardinale et al., 2006, 2008; Grube et al., 2009; Hodkinson and Lutzoni, 2009; Bjelland et al., 2011; Mushegian et al., 2011; Weiss et al., 2011; Sigurbjörnsdóttir et al., 2015; Park et al., 2016). Alphaproteobacteria usually dominate the lichen microbiome, but other taxa such as Actinobacteria, Firmicutes, Acidobacteria, Betaproteobacteria, Deltaproteobacteria, and Gammaproteobacteria are also found. These bacteria can form highly structured, biofilm-like assemblages on fungal surfaces and within the lichen thallus (Grube et al., 2009). The bacterial communities inhabiting the lichen thalli play important roles in the lichen holobiont (the lichen and its microbiome), by contributing to nutrient supply, resistance against biotic and abiotic stresses, production of vitamins and support of fungal and algal growth by the production of hormones, detoxification of metabolites and degradation of senescing parts of the lichen thallus (Grube et al., 2015; Sigurbjörnsdóttir et al., 2015). Thereby, the bacterial part of the lichen holobiont is suggested to contribute to the survival of lichens under extreme and changing environmental conditions.

The composition of associated bacterial communities of lichens may be shaped by intrinsic and extrinsic factors. Among intrinsic factors affecting the lichen microbiome are thallus age (Cardinale et al., 2012b), mycobiont species, and photobiont species (Grube et al., 2009; Weiss et al., 2011; Hodkinson et al., 2012; Wedin et al., 2016; Coleine et al., 2019). The composition of lichen bacterial communities can also be influenced by extrinsic factors such as sunlight exposure and substrate type (Cardinale et al., 2012b; Park et al., 2016), geography and local habitat (Cardinale et al., 2012a; Hodkinson et al., 2012; Printzen et al., 2012; West et al., 2018), altitude (Coleine et al., 2019), drought (Cernava et al., 2019), and arsenic contamination (Cernava et al., 2018). Some lichens can adapt to changing environmental factors by switching photobionts depending on the ecological niche of the photobiont (Domaschke et al., 2013; Rolshausen et al., 2018). Some lichens have also been shown to be able to acclimate to higher temperature by increasing their respiration (Lange and Green, 2005) or net photosynthesis (Colesie et al., 2018). However, not all lichens are able to adapt to changing environments in these ways. Lichens might also be able to acclimate through changes in their associated bacterial communities. This strategy has been demonstrated for several environmental factors, such as drought (Cernava et al., 2019) and arsenic contamination (Cernava et al., 2018). Substrate type is another extrinsic factor that can influence the composition of bacterial communities (Cardinale et al., 2012b). Therefore, changes in C (carbon) or N (nitrogen) availability in the environment, for instance as a result of changes in plant litter quality due to shrubification (McLaren et al., 2017), might be factors altering the structure of the lichen microbiome. However, little is known about the effect of long-term environmental changes on the bacterial communities associated with lichens.

High-latitudes are especially rich in lichen species and biomass (Cornelissen et al., 2007; Nash, 2008), where they make significant contributions to ecosystem functioning (Asplund and Wardle, 2017). Mat-forming lichens such as Cetraroid species contribute to primary production and nutrient cycling, control soil chemistry and water retention (Cornelissen et al., 2007). Currently, climate in high-latitudes warms twice as fast as elsewhere (IPCC, 2019) resulting in increased abundance of shrubs, particularly in the low and sub-Arctic (Elmendorf et al., 2012; Myers-Smith et al., 2019). Direct effects of warming on lichens include changes in C-based secondary compounds (Asplund et al., 2017) and increased biomass (Biasi et al., 2008). Warming also has indirect effects on lichens. In many low and sub-Arctic tundra ecosystems shrubification results in increased shading and greater amounts of litter, which can lead to decreased lichen photosynthesis rates causing a decline in lichen biomass (Nash and Olafsen, 1995; Cornelissen et al., 2001; Elmendorf et al., 2012; Fraser et al., 2014; Alatalo et al., 2017). Yet, the effect of long-term warming on the bacterial communities of lichens in high-latitudes needs to be investigated.

In this study we investigate the total and potentially metabolically active bacterial community of the lichen *Cetraria islandica* (L.) Ach. (English “Iceland moss”) and its response to two decades of warming in open top chambers (OTCs) in an Icelandic sub-Arctic alpine dwarf-shrub heath. *C. islandica* is a mat-forming chlorolichen with foliose thalli and forms a major component of the vegetation in Arctic, sub-Arctic and alpine environments throughout the northern hemisphere (Kärnefelt et al., 1993). 16S rRNA and rDNA sequencing was used to characterize the potentially active and total bacterial community in control plots and OTCs. We also quantified 16S rRNA gene abundance by quantitative PCR to compare the absolute abundance of bacteria in the controls and OTCs. Finally, it was recently demonstrated that N_2_-fixing bacteria could associate with chlorolichens (Almendras et al., 2018). Thus, we also quantified the number of *nifH* genes by quantitative PCR in order to test if the *C. islandica* microbiome could potentially perform N_2_-fixation and how warming influences the abundance of associated N_2_-fixers.

We predicted that long-term warming and the associated increase in tundra shrubs and litter will lead to an increase in heterotrophic, biopolymer-degrading bacterial taxa and a higher incidence of potentially lichenivorous or lichenopathogenic bacteria, such as shown for the plant phyllosphere (Aydogan et al., 2018). Thus, in terms of taxonomic composition, we expected an increase in detritivorous taxa, endosymbionts and pathogens of fungi such as chitinolytic bacteria (Kobayashi and Crouch, 2009), whereas the relative abundance of cold-adapted and facultatively lithotrophic bacteria may decrease.

We also hypothesized that the potentially metabolically active (16S rRNA based) community shows a larger change in richness, diversity and community structure to the warming treatment than the total bacterial community, as has been shown for the effect of drought (Bastida et al., 2017).

## Materials and Methods

### Study Site and Experimental Design

The study site is located in a *Betula nana* heath in the Icelandic central highlands at an altitude of 450 m. According to Köppen’s climate definitions, the sampling site, called Au$\tilde{}\,\partial$kúluhei$\tilde{}\,\partial$i (65°16′N, 20°15′W, 480 m above sea level) is situated in the lower Arctic. The vegetation is characterized as a relatively species-rich dwarf shrub heath, with *B. nana* being the most dominant vascular species and the moss *Racomitrium lanuginosum* and the lichen *C. islandica* as the dominating cryptogam species (Jonsdottir et al., 2005).

Ten OTCs were set up to simulate a warmer summer climate in August 1996 in a fenced area to exclude sheep grazing (Hollister and Webber, 2000; Jonsdottir et al., 2005). The OTCs raise the mean daily temperature by 1–2°C during summer and minimize secondary experimental effects such as differences in atmospheric gas concentration and reduction in ambient precipitation. Control plots were established adjacent to the OTCs, but without any treatment, thus exposing the environment to ambient temperatures. Air temperatures measured in the growing season from 1999 to 2002 at the surface of the cryptogam layer indicated an average increase of 0.7–1.0°C in the OTCs (Jonsdottir et al., 2005). Air temperature measured 10 cm above the moss layer in summer 2016 was on average 1.4°C higher in the OTC than in the control plot of one of the plot pairs (*t* = −8.2, *P* < 0.001) (Supplementary Table 1). Relative humidity measured in the same plot pair and period was 3% lower in the OTC than in the control plot (*t* = 26.9, *P* < 0.001) (Supplementary Table 1). Temperatures on the moss surface measured in all OTCs and control plots from mid-August 2018 to mid-June 2019 were on average 0.22°C higher in the OTCs compared to the control plots (*t* = −16.4, *P* < 0.001) (Supplementary Table 1).

The response of the vegetation was monitored by a detailed vegetation analysis after peak biomass at a few year intervals using the point intercept method following standard protocols of the International Tundra Experiment (ITEX: 100 points per plot, all hits (intercepts) per species recorded in each point through the canopy; relates to biomass) (Jonsdottir et al., 2005). In 2014, the abundance of *B. nana* was on average 2.5 times larger in the OTCs than in the control plots and litter was 2.7 times more abundant in the OTCs than in the control plots (Jónsdóttir, unpublished data). In this study we use these data on abundance (total number of hits per plot) of *B. nana* and litter to test the effect of vegetation change on the richness, diversity, community structure of the lichen associated bacterial community as well 16S rRNA and *nifH* gene abundance.

Per warmed (OTC) and control plot, the upper parts (2 × 2 cm) of five lichen thalli were randomly selected and collected with sterile tweezers. The samples were immediately soaked in RNAlater (Ambion) to prevent RNA degradation and kept cool until storage at −80°C. The lichen samples were collected in June 2017.

### RNA and DNA Extraction and Sequencing

Prior to RNA and DNA extraction, the samples were washed with RNase free water to get rid of soil particles and RNAlater and ground for six minutes using a Mini-Beadbeater and two sterile steel beads. RNA and DNA were extracted simultaneously using the RNeasy PowerSoil Total RNA Kit (Qiagen) and the RNeasy PowerSoil DNA Elution Kit (Qiagen), following the manufacturer’s instructions. DNA and RNA concentrations were measured with a Qubit Fluorometer (Life Technologies) and purity was assessed with a NanoDrop (NanoDrop Technologies) and integrity by Bioanalyzer (Agilent Technologies). cDNA was synthesized using the High-Capacity cDNA Reverse Transcription Kit (Thermofisher) following the manufacturer’s instructions and quantified on a Qubit Fluorometer (Life Technologies). From the 100 DNA and 100 cDNA samples, we selected 48 DNA samples (24 from each treatment) and 48 cDNA samples (24 from each treatment) for sequencing based on the RNA and DNA quantity and quality. Library preparation and sequencing of the V3–V4 region of the 16S rRNA gene on an Illumina MiSeq platform was performed by Macrogen (Seoul), using the standard Illumina protocol. The primer pair 337F/805R and the same PCR condition described in Klindworth et al. (2013) were used.

### Sequence Processing

In order to obtain high-resolution data, we processed the raw sequences using the DADA2 pipeline (Callahan et al., 2016, 2017). Hereby, sequences are not clustered into operational taxonomic units (OTUs), but exact sequences or amplicon sequence variants (ASVs). Forward reads were truncated at 260 bp and reverse reads at 250 bp. Assembled ASVs were assigned taxonomy against the SILVA_132 database (Quast et al., 2013) using the Ribosomal Database Project (RDP) naïve Bayesian classifier (Wang et al., 2007) in DADA2. We discarded samples with less than 10,000 non-chimeric sequences and/or less than 50 ASVs. We removed ASVs assigned to chloroplasts and mitochondria, singletons, doubletons and ASVs occurring in only one sample. In total, for 82 samples, 1,954 ASVs remained. The data were normalized using cumulative-sum scaling (CSS) (Paulson et al., 2013) to account for uneven sequencing depths.

The 16S rDNA based community is hereafter sometimes referred to as the DNA based community and the 16S rRNA (cDNA) based community is hereafter referred to as the cDNA based community. We interpret the cDNA based community as the “potentially metabolically active bacterial community,” acknowledging that 16S rRNA is not a direct indicator of activity but rather protein synthesis potential (Blazewicz et al., 2013).

### Quantitative Real-Time PCR of nifH and 16S rRNA Genes

We used all 100 DNA extractions (50 replicates per treatment) for quantification of *nifH* and 16S rRNA genes, which was performed by quantitative PCR (Corbett Rotor-Gene) using the primer set PolF/PolR and 341F/534R, respectively (Poly et al., 2001). The specificity of the *nifH* primers for our samples was confirmed by SANGER sequencing of 10 clone fragments. Standards for *nifH* reactions were obtained by amplifying one cloned *nifH* sequence with flanking regions of the plasmid vector (TOPO TA cloning Kit, Invitrogen). Standard curves were obtained by serial dilutions (E = 0.9–1.1, *R*^2^ = > 0.99 for all reactions). Each reaction had a volume of 20 μL, containing 1× QuantiFast SYBR Green PCR Master Mix (Qiagen), 0.2 μL of each primer (10 μM), 0.8 μL BSA (5 μg/μL), 6.8 μL RNase free water, and 2 μL template. The cycling program was 5 min at 95°C, 30 cycles of 10 s at 95°C and 30 s at 60°C.

### Statistical Analysis

Statistical analyses were conducted in R version 3.6.1. Richness (number of ASVs) and Shannon diversity were calculated with the R packages “vegan” (Oksanen et al., 2013) and “phyloseq” (McMurdie and Holmes, 2013).

Differences in 16S rRNA and *nifH* gene abundance, ASV richness and Shannon diversity between the treatments and the DNA and cDNA were assessed with Bayesian (Markov chain Monte Carlo) generalized linear models using the R package “MCMCglmm” (Hadfield, 2010) with treatment as a fixed and plot as a random factor to take into account the variation caused by pseudoreplication. We considered differences significant if the modeled 95% confidence intervals did not overlap. In addition to the effect of treatment, we included *B. nana* and litter abundance in these generalized linear models. The effect of *B. nana* and/or litter abundance was considered significant if 95% High Posterior Density Credible Interval (95% CrI) were not overlapping zero.

Distances between the community composition of the control and OTC samples were based on Bray-Curtis distances. The effect of the treatment, *B. nana* abundance and litter abundance on the bacterial community composition was tested by permutational-MANOVA (PERMANOVA) (Anderson, 2001) analysis of the Bray-Curtis distance matrices using the *adonis* function in the R package “vegan” with plot as strata. Principal coordinate analysis was used to ordinate the Bray-Curtis distance matrices and to visualize the relationships between samples from OTC and control plots.

For the comparisons of relative abundances of taxa on phylum, class and order level between the warmed and the control samples, pseudoreplicates were averaged by the OTC or control plot they originated from. These average relative abundances were then compared using Wilcoxon rank-sum tests.

We used two methods to determine taxa sensitive to warming. For both methods, we used the average abundances of ASVs in each plot. First, differential abundance of bacterial ASVs between warmed and control samples was assessed using DESeq2 (Love et al., 2014) on the non-CSS normalized datasets with the R package “DESeq2” (Love et al., 2014). The adjusted *P*-value cut-off was 0.1 (Love et al., 2014). Differential abundance analysis only uses ASVs present in both the OTC and control samples. The second method we used to find taxa sensitive to warming, was indicator species analysis. To find bacterial taxa indicative for the warming or the control treatment, correlation-based indicator species analysis was done with all possible site combinations using the function *multipatt* of the R package “indicSpecies” (De Caceres and Legendre, 2009) based on 10^3^ permutations. The indicator species analysis takes into account ASVs present in both OTC and control samples, but also ASVs present in only one of the treatments. We combined results of the DESeq2 and indicator species analysis for a final list of ASVs sensitive to warming.

For visualizations of the data, we showed all samples when we could account for pseudoreplication (Bayesian generalized linear models and Permanovas) and we showed plot averages when we compared between the control and warmed treatment (Wilcoxon rank-sum tests).

## Results

### Effect of OTC Treatment on ASV Richness, Diversity and Community Structure

**FIGURE 1:**
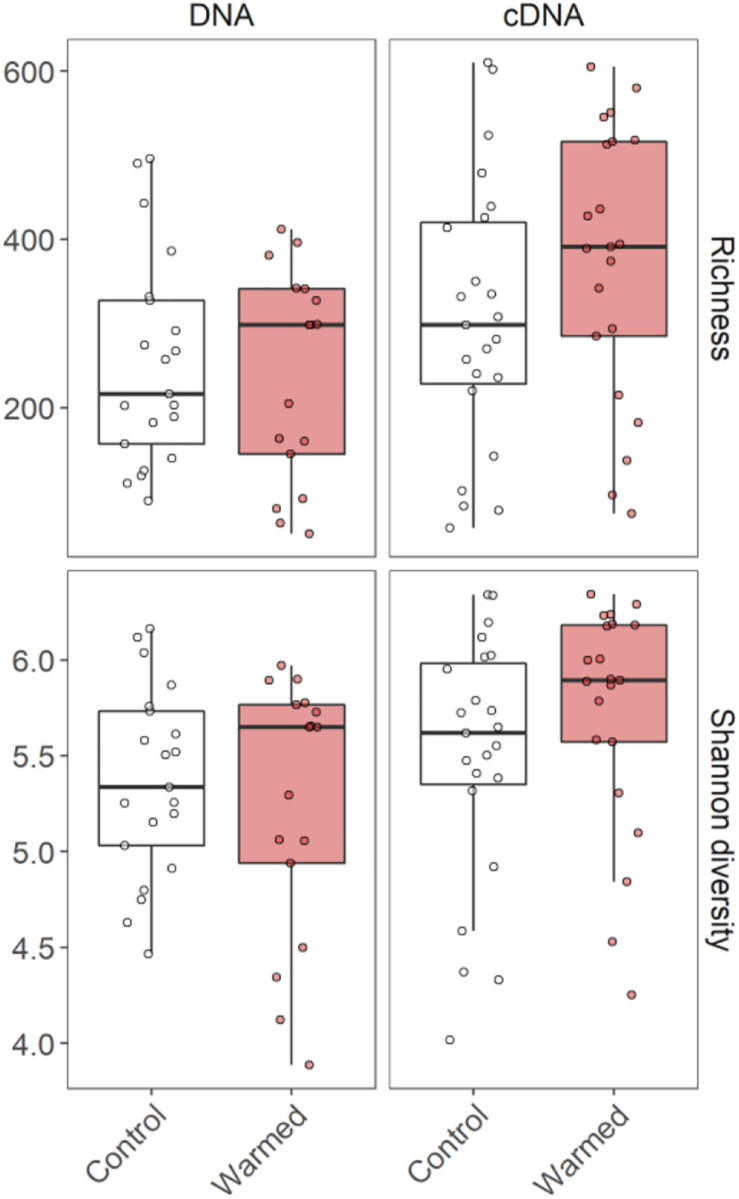
ASV richness and Shannon diversity of the DNA and cDNA-based bacterial communities associated with the lichen *C. islandica* in control and warmed samples. *N* = 82, *n* = 21 for DNA control, *n* = 17 for DNA warmed, *n* = 23 for cDNA control and *n* = 21 for cDNA warmed.

The ASV richness and Shannon diversity of the bacterial communities associated with *C. islandica* were not significantly affected by the warming treatment (Figure 1 and Supplementary Figures 1–4). However, we found that *B. nana* abundance tended to positively influence the cDNA-based bacterial richness and Shannon diversity (Supplementary Figures 3, 4). A significant difference was found for the richness, which was higher for the cDNA-based bacterial community than the DNA-based community in the warmed treatment (Supplementary Figure 7). The cDNA-based Shannon index tended to be higher than the DNA-based Shannon index (Figure 1 and Supplementary Figure 5).

**FIGURE 2:**
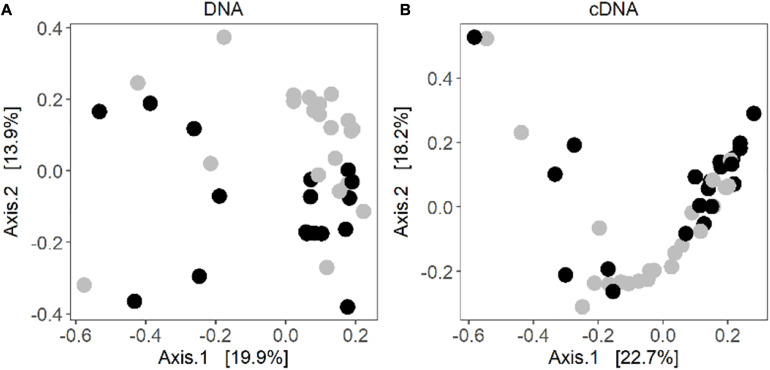
Principal coordinate analysis of Bray-Curtis distances of the **(A)** DNA-based community composition and **(B)** cDNA-based community composition of the lichen *C. islandica*. Warmed samples are represented as black circles and control samples as gray circles. *N* = 28, *n* = 21 for DNA control, *n* = 17 for DNA warmed, *n* = 23 for cDNA control and *n* = 21 for cDNA warmed.

Some level of clustering between the control and warmed samples could be observed in the principal coordinate analysis (Figure 2). Based on the results of a PERMANOVA, the warmed lichen associated bacterial communities were significantly different from the communities in the control samples (DNA: *R*^2^ = 0.07 and *P* = 0.001; RNA: *R*^2^ = 0.07 and *P* = 0.005) (Supplementary Tables 2, 3). In addition, litter abundance was associated with variation in the cDNA-based bacterial community (Permanova: *R*^2^ = 0.04 and *P* = 0.05).

### Effect of OTC Treatment on the Taxonomic Composition and Abundance of the *C. islandica* Bacteriota

**FIGURE 3:**
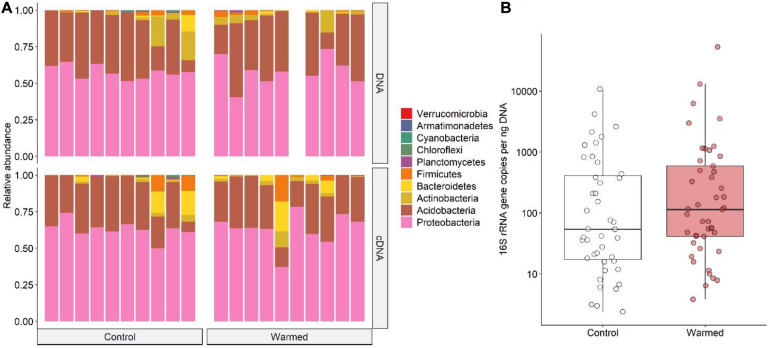
**(A)** Barplots of the bacterial community composition associated with the lichen *C. islandica* in the control and warmed plots. Data are presented at the phylum level for DNA and cDNA. Bars represent pooled samples from each separate plot. **(B)** 16S rRNA gene abundance per ng extracted DNA in control (white) and warmed (red) samples. *N* = 88, *n*_warmed_ = 45, *n*_control_ = 43.

**FIGURE 4:**
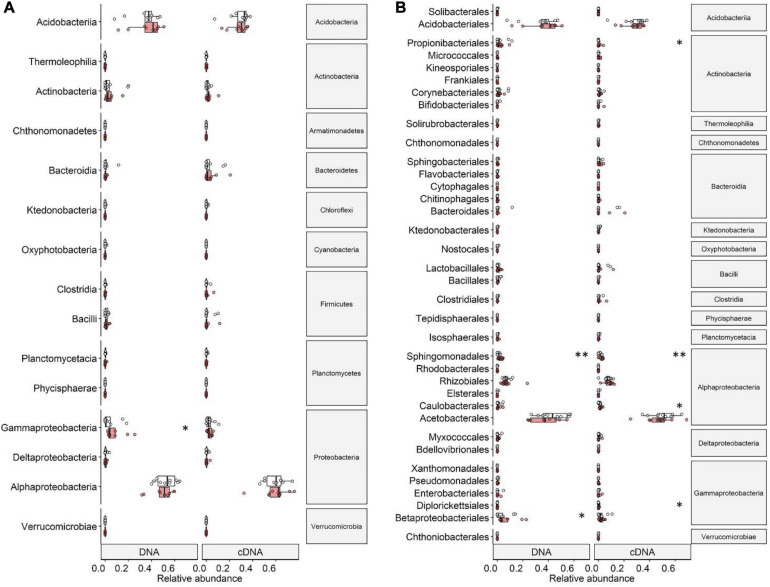
Relative abundances of **(A)** classes and **(B)** orders of DNA- and cDNA-based bacterial communities associated with the lichen *C. islandica* in control (white) and warmed (red) samples. Points indicate average relative abundance values per control or warmed plot. Boxplots represent minimum values, first quartiles, medians, third quartiles, and maximum values. Significance levels (^∗^*p* < 0.05 and ^∗∗^*p* < 0.01) are based on Wilcoxon rank-sum tests.

The bacterial community found associated with the lichen *C. islandica* is described at the phylum level (Figure 3A) and at the class and order level (Figure 4). No clear differences were found for the relative abundance at the phylum level between the control and warmed treatment for the total bacteria community (Figure 3A). Similarly, we did not detect differences between the control and warmed treatment in the cDNA-based bacterial community at the phylum level (Figure 3A). The total bacterial community was dominated by Proteobacteria and Acidobacteria (DNA: 58 and 34% average relative abundance across all control and warmed samples, for Proteobacteria and Acidobacteria, respectively). Proteobacteria and Acidobacteria were also the main phyla in the cDNA-based bacterial community (cDNA: 63 and 29%, respectively) (Figure 3A). At lower taxonomic level, the orders Acetobacterales and Acidobacteriales were the dominant taxa (DNA: 44%; cDNA: 51%, DNA: 34%; cDNA 29%, Figure 4A). Within the acidobacterial family Acetobacteraceae, about 14% could not be assigned to a genus (Supplementary Figure 7). The most abundant genera in the cDNA and DNA-based bacterial communities were the proteobacterial genera *Acidiphilium* (DNA: 8%, cDNA: 11%) and *Endobacter* (DNA: 19%, cDNA: 20%) and the acidobacterial genera *Bryocella* (DNA: 10%, cDNA: 9%) and *Granulicella* (DNA: 15%, cDNA: 11%) (Supplementary Figure 7).

A total of 295 ASVs were only detected in the cDNA-based samples and not in the DNA-based samples. These taxa belonged to abundant genera such as *Endobacter* and *Acidiphilium* (Supplementary Tables 4, 5). Genera that were exclusively found in the cDNA-based community were *Lactococcus*, *Lachnospiraceae NK4A136* group, *Ktedonobacter*, *Methylovirgula*, *Frigoribacterium*, *Amnibacterium*, *Rhizobacter*, *Telmatobacter*, *Kineosporia*, and *Acidiphilium* (Supplementary Tables 4, 5).

The bacterial load was on average 671 and 1944 16S rRNA copies per ng DNA for control and warmed plots, respectively (Figure 3B). However, no differences could be found between the overall 16S rRNA gene copy numbers, or *nifH* gene copy numbers in the control and warmed plots (Figure 3B and Supplementary Figures 8, 9). Nevertheless, *B. nana* abundance positively affected both *nifH* and 16S rRNA gene copy numbers, whereas litter abundance negatively affected *nifH* gene copy numbers (Supplementary Figures 8, 9).

In the DNA-based samples, the only proteobacterial class found significantly affected by warming were the Gammaproteobacteria with an increase of 50% in the warmed samples (Wilcoxon rank-sum test, *P* = 0.033; Figure 4A). Most of this increase was due to an increase in relative abundance of the order Betaproteobacteriales (*P* = 0.046) (Figure 4B). The order Sphingomonadales (Alphaproteobacteria) increased by a factor of 2.5 in relative abundance (*P* = 0.009) (Figure 4B). In the cDNA-based bacterial community, we did not detect differences in relative abundance on Proteobacterial class level between the control and warmed treatment (Figure 4A). Changes were found on order level with the alphaproteobacterial order Caulobacterales being twice as abundant in the cDNA-based bacterial community in the warmed plots (*P* = 0.038) than in the control plots. Similarly, the order Sphingomonadales was 2.7 times more active in the warmed treatment (*P* = 0.007) (Figure 4B). The order Diplorickettsiales (Gammaproteobacteria) was four times as abundant in the cDNA-based bacterial community in the warmed plots (*P* = 0.040) (Figure 4B).

While the phylum Actinobacteria was not among the most common phyla (DNA: 4%, cDNA: 2%), its order Propionibacteriales was 20 times as abundant in the cDNA-based bacterial community in the warmed plots compared to the control plots (*P* = 0.040) (Figure 4B).

### Effect of OTC Treatment on the Relative Abundance of Bacterial ASVs

For the DNA-based communities, we detected 61 ASVs with a higher relative abundance in the warmed samples with a total relative abundance of 1% (Supplementary Table 4). We detected 96 ASVs with a lower relative abundance in the warmed samples compared to the control samples making up 1.7% of the total abundance (Supplementary Table 4). For the cDNA-based bacterial communities, we detected 190 ASVs with a higher relative abundance in the warmed samples (2.12%) and 77 ASVs with a lower relative abundance in the warmed samples compared to the control samples (0.9%) (Supplementary Table 5).

Of the ASVs only detected in cDNA-based bacterial community, 14 ASVs had a higher relative abundance in the warmed plots. All these rare ASVs belonged to the Proteobacteria, except one ASV that was classified as Bacteroidetes.

**FIGURE 5:**
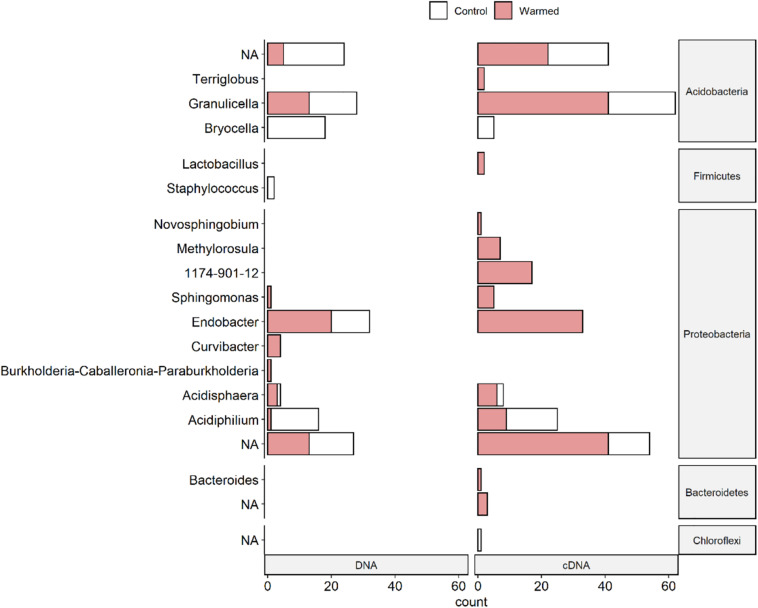
Number of ASVs (amplicon sequence variants) per genus sensitive to warming for DNA and cDNA-based bacterial communities associated with the lichen *C. islandica*. Sensitivity to warming was determined by differential abundance analysis (DESeq2) and indicator species analysis. ASVs not assigned to genus level are called “NA.” The number of ASVs indicative for the OTC (warmed) treatment is indicated in red and the number of ASVs indicative for the control treatment is indicated in white.

Amplicon sequence variants within the Proteobacteria showed mainly increased relative abundance in the warmed samples (Figure 5 and Supplementary Tables 4, 5). Only ASVs classified under the genus *Acidiphilium* had more often a lower relative abundance in the warmed samples as well as a few ASVs of the genera *Acidisphaera* and *Endobacter*. In the cDNA-based samples, more proteobacterial ASVs with increased relative abundances under warming were detected than in the DNA-based samples.

Acidobacterial ASVs showed mixed differences between the control and warmed samples (Figure 5 and Supplementary Tables 4, 5). ASVs of the genus *Bryocella* were less abundant under warming in both the DNA- and cDNA-based samples. ASVs of the genus *Granulicella* were equally more and less abundant in the warmed DNA-based samples, but had more often higher relative abundances in the warmed cDNA-based samples (Figure 5 and Supplementary Tables 4, 5).

## Discussion

We assessed the effect of long-term (20 years) warming by OTCs on the bacterial community composition associated with the lichen *C. islandica* in an Icelandic sub-Arctic alpine dwarf-shrub heath. The community was dominated by Acidobacteria and Proteobacteria in both total and potentially active bacterial communities in both control and warmed plots. Warming did not induce compositional or structural changes at higher taxonomical levels. Nevertheless, we found indications of multiple warming-induced shifts in the community composition at the class, order and ASV levels. The most prominent increases in relative abundance were found in several genera belonging to the Proteobacteria. Our results illustrate that the long-term warming treatment affects the bacterial community composition of the lichen symbiosis at fine taxonomical levels.

### The Bacterial Community of *Cetraria islandica*

While the dominance of the class Alphaproteobacteria has been described as a general characteristic of lichen bacterial communities (Cardinale et al., 2008; Weiss et al., 2011; Printzen et al., 2012), a striking feature of the *C. islandica* microbiome is the strong dominance of the family Acetobacteriaceae (Alphaproteobacteria). While the presence of Acetobacteriaceae in lichens has been observed before, notably in the reindeer lichen *Cladonia arbuscula* (Cardinale et al., 2008), this strong dominance does seem unusual. Indeed, the Rhizobiaceae, often held to be the dominant Alphaproteobacteria in lichens (Weiss et al., 2011; Hodkinson et al., 2012), only make up a minor part of the *C. islandica* bacteriome. The second dominant group in the *C. islandica* bacteriome are members of the family Acidobacteriaceae (Acidobacteria). Even at the genus level, the *C. islandica* bacteriome is surprisingly homogeneous, with approximately half of the reads being assigned to only four genera, the acetobacterial genera *Endobacter* and *Acidiphilium*, and the acidobacterial genera *Granulicella* and *Bryocella*. This pronounced dominance of presumptively acidophilic taxa is noteworthy. Acidobacteria were also reported earlier in living parts of bog and tundra (Pankratov, 2012) and lichens in Alpine soil crusts (Muggia et al., 2013). The presence of acidophilic taxa may be explained by organic acid secondary metabolites produced by *C. islandica*, such as protolichesterinic and fumaroprotocetraric acids (Xu et al., 2018).

Another feature of the *C. islandica* microbiota is the difference between the potentially metabolically active and total community. The richness of the potentially active bacterial communities was higher compared the to total bacterial communities. One possible explanation for this is that the detection of taxa in 16S rRNA sequences, but not in 16S rDNA sequences, can occur when rare taxa have a high metabolic potential. The occurrence of these “phantom taxa” could be a result of cDNA synthesis errors that do not occur in the rDNA samples, but are introduced in the rRNA sequences. Another explanation could be variation in metabolic activity among taxa (Campbell et al., 2011; Baldrian et al., 2012; Klein et al., 2016; Jia et al., 2019). Rare taxa have been observed to be disproportionally active compared to abundant members (Jones and Lennon, 2010) and thereby might contribute more to ecosystem functioning than one would expect based on their abundance (Jousset et al., 2017). The rare bacterial taxa of *C. islandica* were mostly composed of not assigned genera and members of the genera *Endobacter*, *Acidiphilium*, *Lactococcus*, *Mucilaginibacter*, and *Bacteroides*. The fermenting bacteria from the genus *Lactococcus* have been described before in a bioreactor as being rare while having high potential activity levels (Lawson et al., 2015).

N_2_-fixation is an important process in N-limited tundra ecosystems and previous work has shown that biocrust chlorolichens can show significant nitrogenase activity (Torres-Cruz et al., 2018). As *C. islandica* is a chlorolichen and does not have N_2_-fixing Cyanobacteria as a photobiont, this raises the question if other taxa could be N_2_-fixers. The N_2_-fixing capability of *C. islandica* is unknown, but the presence of *nifH* genes indicates that potential N_2_-fixers are present in the *C. islandica* bacterial community. Indeed, we detected putative N_2_-fixers such as *Curvibacter* (Ding, 2004) and members of the Burkholderiaceae. On the other hand, lichens might also obtain nutrients dissolved in precipitation or through runoff from taller vegetation, or via a moisture gradient resulting in upward movement of soil moisture and dissolved nutrients (Longton, 1992). Nevertheless, our data indicate that the indirect effect of warming through changes in litter and *B. nana* abundance can influence the abundance of N_2_-fixing bacteria. More studies on the N_2_-fixation capabilities of chlorolichens in tundra ecosystems are necessary to elucidate their role as N_2_-fixers.

The *C. islandica*-associated microbiota was found to be markedly different to that of the moss *Racomitrium lanuginosum* which was studied in the same warming experiment (Klarenberg et al., 2019), further supporting the host-specific selection of bacteria from the environment and symbiotic nature of both bryophyte and lichen holobionts proposed in the recent literature (Aschenbrenner et al., 2016; Holland-Moritz et al., 2018). Specifically, we found that *C. islandica* harbored a less rich and diverse bacterial community than *R. lanuginosum*, and the microbiota composition was profoundly different. Whereas the moss was dominated by the genera *Haliangium*, *Acidiphilium*, *Nostoc*, *Conexibacter*, *Granulicella*, *Solibacter*, and *Bryobacter*, the lichen was dominated by a few genera (*Bryocella*, *Granulicella*, *Acidiphilium*, and *Endobacter*) as reported herein. The same difference between the bacterial diversity of a lichen and a moss was shown by Aschenbrenner et al. (2017).

### The Effect of OTC Warming on Bacterial Richness, Diversity and Community Structure

While we did not see any significant changes in richness or diversity of the bacterial community with warming, the warmed bacterial community structure significantly differed from the control community, both for the total as well as the potentially active communities. Overall, the potentially active community tended to be more affected by warming than the total bacterial community. For instance, more indicator taxa were found in the potentially active community and many more of these indicators were found in the warmed treatment. In addition, indirect effects of warming via shrubification and litter modification were found to affect the bacterial community. A positive effect of *B. nana* abundance was found on the richness and diversity of the potentially active community as well as on 16S rRNA and *nifH* gene abundance. Litter abundance was positively associated with *nifH* gene abundance and with the structure of the potentially active bacterial community.

At a coarse taxonomic level, the bacterial community structure was quite similar between the control and warmed treatment. One possible explanation for the similarity between the richness, diversity and composition of the warmed and control lichen bacterial communities could be that over long periods of warming bacterial communities acclimatize (Bradford et al., 2008; Crowther and Bradford, 2013; Romero-Olivares et al., 2017). Nonetheless, at lower taxonomic levels (class, order, and ASV) we detected differences in relative abundances. Shifts in individual taxa can affect microbe–microbe and microbe–host interactions and potentially change functionality or stability of the lichen-associated bacterial communities and thereby influence host health and ecosystem functioning, as proposed for plant–microbiomes (Agler et al., 2016; van der Heijden and Hartmann, 2016; Aydogan et al., 2018).

Long-term warming decreased the relative abundance of ASVs belonging to the Acidobacterial genera *Granulicella* and *Bryocella* and the alphaproteobacterial genus *Acidiphilium* in the total bacterial communities. *Acidiphilium* and *Granulicella* have been observed in other lichen microbiomes (Bates et al., 2011; Pankratov, 2012; Park et al., 2016; Aschenbrenner et al., 2017). These genera are chemoorganotrophic or chemolithotrophic and might thus survive on C sources present in the lichen thallus. *Granulicella* encompasses several acidophilic, cold-adapted species described from tundra soil isolates (Männistö et al., 2012). It has hydrolytic properties such as the ability to degrade chitin (Pankratov and Dedysh, 2010; Pankratov, 2012; Park et al., 2016; Belova et al., 2018), which suggest a role for these bacteria in the degradation of senescing lichen thalli. While some ASVs of the genus *Granulicella* decreased in relative abundance, the increased potential activity might result in increased degradation of dead lichen material. In contrast, the decreased relative abundance and potential activity of *Bryocella* and *Acidiphilium* might result in slower degradation of dead lichen material. The genus *Acidiphilium* showed an increase in relative abundance in a moss microbiome in the same warming experiment (Klarenberg et al., 2019). This suggests that the responses of microbiome components to environmental change are at least in part dependent upon host vegetation identity rather than constituting a direct response of the bacteria themselves to extrinsic environmental factors. Thus leading to different outcomes for the various microbiomes within the same environment.

The alphaproteobacterial genera *Acidisphaera*, *Sphingomonas*, and *Endobacter* showed an increased relative abundance and potential activity with warming. *Sphingomonas* and *Acidisphaera* have been identified in other lichen bacterial communities (Cardinale et al., 2008). *Endobacter* is a poorly characterized genus of which only one species has been described (Ramirez-Bahena et al., 2013). *Acidisphaera* is chemoorganotrophic and contains bacteriochlorophyll (Hiraishi et al., 2000). *Sphingomonas* is known for its ability to degrade plant biomass, the utilization of recalcitrant matter in oligotrophic environments, and the use of sulfonated compounds as sources of C and sulfur (Aylward et al., 2013), which may be linked to the increase in litter abundance. The increase in relative abundance and potential activity of these genera in the warmed conditions might enhance C and nutrient availability in the lichen thalli.

Overall, all genera that dominated the bacterial community of *C. islandica* contained ASVs that were affected by the warming treatment in their relative abundances and potential metabolic activity. The genera that were affected in relative abundance are likely to play roles in nutrient recycling and supply in the lichen symbiosis. As most of these ASVs increased in relative abundance with warming, nutrient turnover in the lichen might be accelerated.

OTCs have been deployed to study warming effects in a wide range of ecosystems and plant responses correspond well to responses to natural climate warming (Hollister and Webber, 2000). We have shown that the OTC treatment leads to changes in the composition of the bacterial community associated with the lichen *C. islandica*, and that part of this change could be attributed to the increase in *B. nana* and litter abundance in the OTCs. This secondary effect of the OTC treatment may shield radiation from reaching the lichen layer or soil and thereby reduce the warming effect of the OTCs (Bokhorst et al., 2013), reducing the amount of PAR reaching the lichen and potentially affecting the bacterial communities. The increase in *B. nana* leaf litter may also increase C turnover as it is easily decomposable (McLaren et al., 2017) and thereby influence the lichen bacterial community. It should be noted that multiple caveats can be associated with the use of OTCs. Snow trapped in OTCs can increase temperatures at the soil surface, but at the same time decrease photosynthetically active radiation (Bokhorst et al., 2013). The walls of the OTCs may act as a barrier for new species to arrive (Richardson et al., 2000), even though it is unknown how important this side effect of the OTCs is on microbial communities. In addition, temperature-induced changes in lichen traits such as thallus nutrient content, as well as soil organic matter content, and soil moisture are environmental factors that could potentially influence the lichen bacterial community composition. Warming could also affect the secondary metabolites of the lichen (Asplund et al., 2017) and thereby alter the composition of the lichen microbiota.

In conclusion, we found that the bacterial community of *C. islandica* was dominated by acidophilic taxa and harbored rare, but potentially active taxa. Our results also showed that twenty years of warming and an increase dwarf-shrub and litter abundance can lead to changes in lichen bacterial communities at a fine taxonomic level as well as richness and diversity. The lichen microbiome plays an important role in the growth of lichens and climate-driven changes in the lichen microbiota, irrespective of whether they are due to direct or indirect effects of climate change, might affect decomposition of lichens and thereby nutrient cycling in sub-Arctic ecosystems.

## Data Availability Statement

The original contributions presented in the study are publicly available. This data can be found in NCBI, under accession PRJEB37116.

## Author Contributions

IJ, IK, and OV designed the study. IK conducted the sampling, the laboratory work, the bioinformatics processing, and the statistical analysis. CK performed the qPCR measurements. IK wrote the manuscript with contributions from OV, IJ, CK, and DW. All authors contributed to the article and approved the submitted version.

## Conflict of Interest

The authors declare that the research was conducted in the absence of any commercial or financial relationships that could be construed as a potential conflict of interest.
