# Supplementary material for: The Total and Active Bacterial Community of the Chlorolichen Cetraria islandica and Its Response to Long-Term Warming in Sub-Arctic Tundra
Source: Front Microbiol. 2020 Dec 18;11:540404. doi: 10.3389/fmicb.2020.540404 (PMC7775390; doi:10.3389/fmicb.2020.540404)
Supplement: Supplementary file 1 [file Data_Sheet_1.DOCX]

Supplementary Material

# Supplementary Figures and Tables

## Supplementary Figures

# Supplementary Figure 1 Fixed effect structure of the linear mixed-effect model testing the effect of treatment, *Betula nana* abundance and litter abundance on the DNA-based richness. Non-overlapping 95% High Posterior Density Credible Interval (95% CrI) are used to detect significant differences between effects. Parameters with 50% CrI overlapping 0 are indicated by open circles. Parameters with 50% CrI not overlapping 0, but with 95% CrI overlapping 0 are indicated by closed black circles. Thick lines represent 50% CrI and thin lines represent 95% CrI.

#
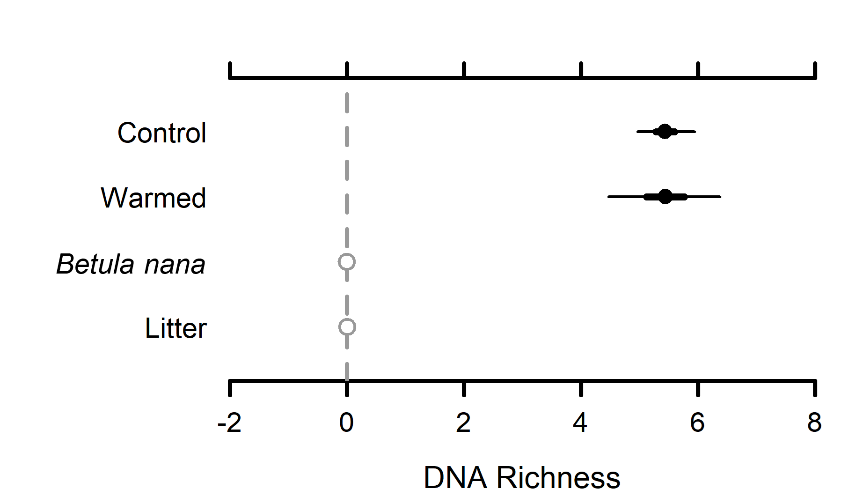


**Supplementary Figure 2** Fixed effect structure of the linear mixed-effect model testing the effect of treatment, *Betula nana* abundance and litter abundance on the DNA-based Shannon diversity. Non-overlapping 95% High Posterior Density Credible Interval (95% CrI) are used to detect significant differences between effects. Parameters with 50% CrI overlapping 0 are indicated by open circles. Parameters with 50% CrI not overlapping 0, but with 95% CrI overlapping 0 are indicated by closed black circles. Thick lines represent 50% CrI and thin lines represent 95% CrI.


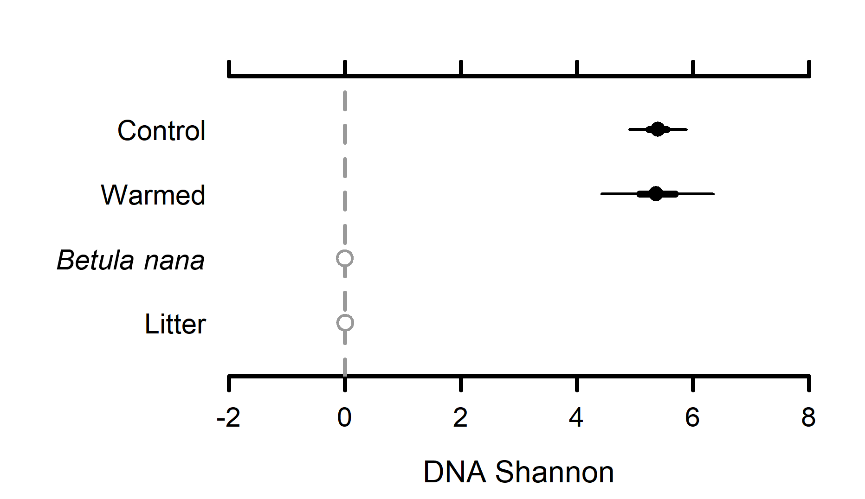


**Supplementary Figure 3** Fixed effect structure of the linear mixed-effect model testing the effect of treatment, *Betula nana* abundance and litter abundance on the cDNA-based richness. Non-overlapping 95% High Posterior Density Credible Interval (95% CrI) are used to detect significant differences between effects. Parameters with 50% CrI overlapping 0 are indicated by open circles. Parameters with 50% CrI not overlapping 0, but with 95% CrI overlapping 0 are indicated by closed black circles. Thick lines represent 50% CrI and thin lines represent 95% CrI.


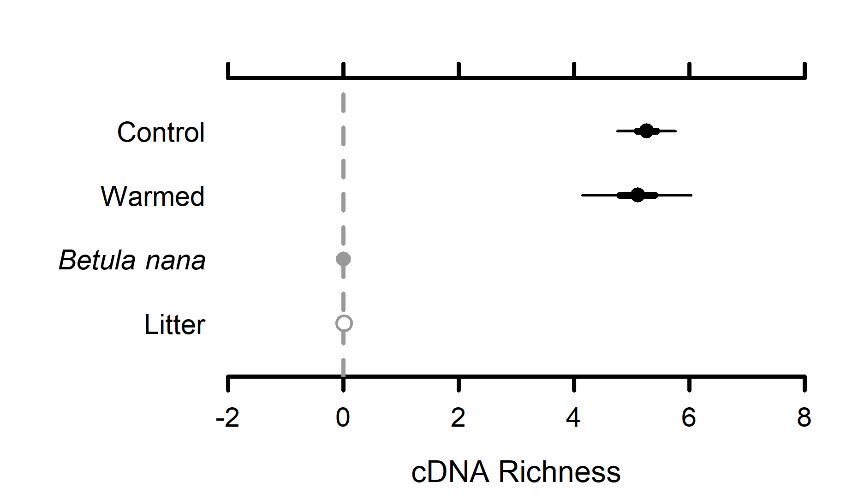


**Supplementary Figure 4** Fixed effect structure of the linear mixed-effect model testing the effect of treatment, *Betula nana* abundance and litter abundance on the cDNA-based Shannon diversity. Non-overlapping 95% High Posterior Density Credible Interval (95% CrI) are used to detect significant differences between effects. Parameters with 50% CrI overlapping 0 are indicated by open circles. Parameters with 50% CrI not overlapping 0, but with 95% CrI overlapping 0 are indicated by closed black circles. Thick lines represent 50% CrI and thin lines represent 95% CrI.


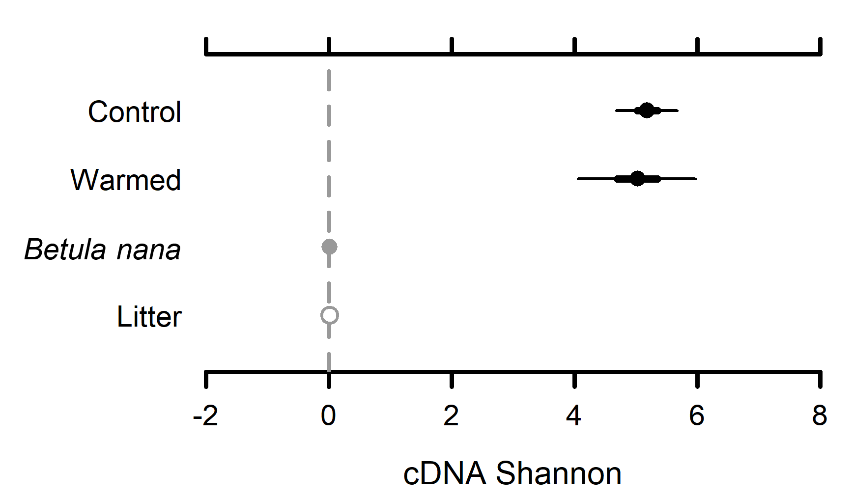


**Supplementary Figure 5** Fixed effect structure of the linear mixed-effect model testing the effect material (DNA vs cDNA) on the ASV richness. Non-overlapping 95% High Posterior Density Credible Interval (95% CrI) are used to detect significant differences. Parameters with 50% CrI overlapping 0 are indicated by open circles. Parameters with 50% CrI not overlapping 0, but with 95% CrI overlapping 0 are indicated by closed black circles. Thick lines represent 50% CrI and thin lines represent 95% CrI.


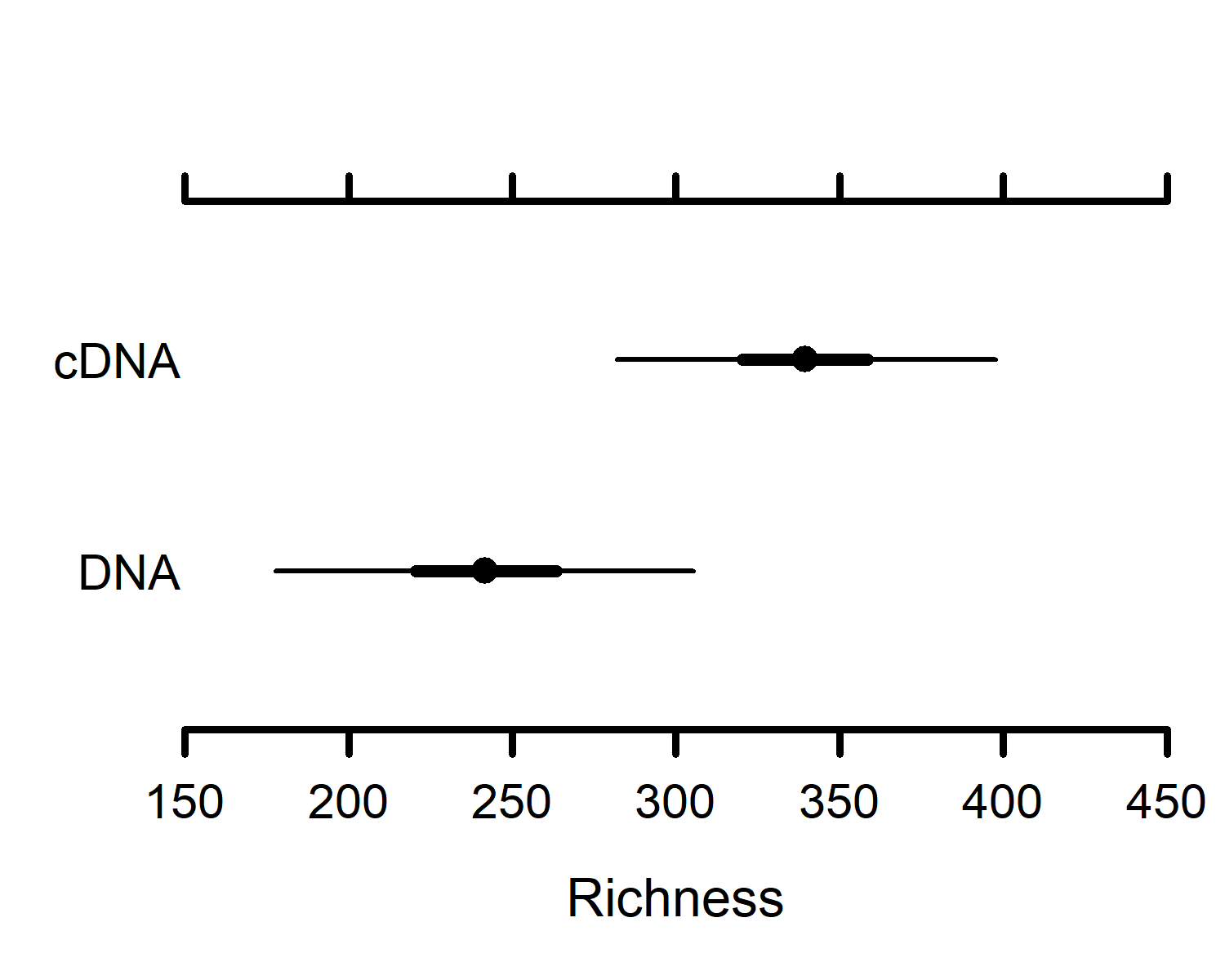


**Supplementary Figure 6** Fixed effect structure of the linear mixed-effect model testing the effect material (DNA vs cDNA) on the Shannon diversity. Non-overlapping 95% High Posterior Density Credible Interval (95% CrI) are used to detect significant differences. Parameters with 50% CrI overlapping 0 are indicated by open circles. Parameters with 50% CrI not overlapping 0, but with 95% CrI overlapping 0 are indicated by closed black circles. Thick lines represent 50% CrI and thin lines represent 95% CrI.


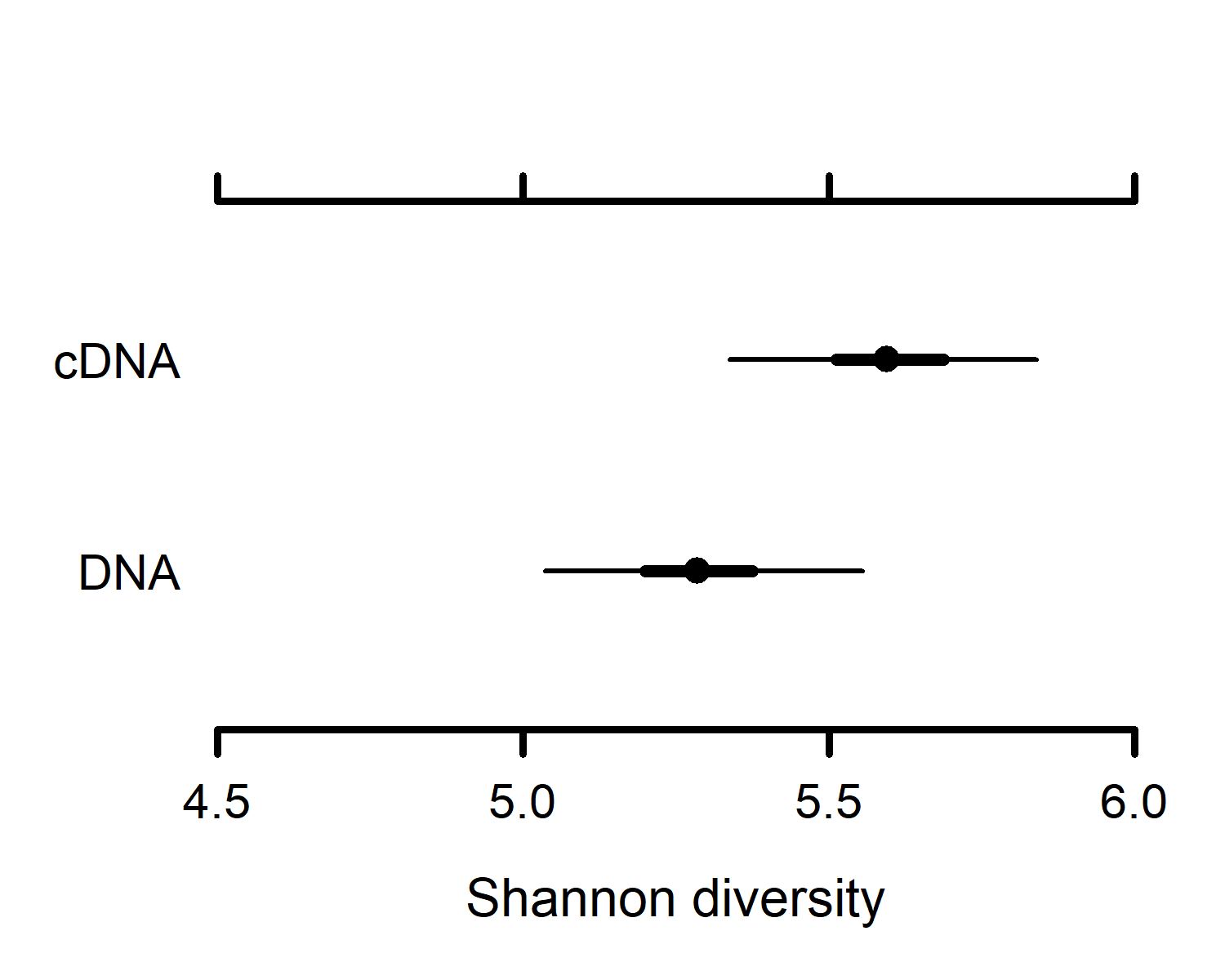


**Supplementary Figure 7** Relative abundances of genera of DNA- and cDNA-based bacterial communities associated with the lichen *Cetraria islandica* in control (white) and warmed (red) samples. Points indicate average relative abundance values per control or warmed plot. Boxplots represent minimum values, first quartiles, medians, third quartiles and maximum values.


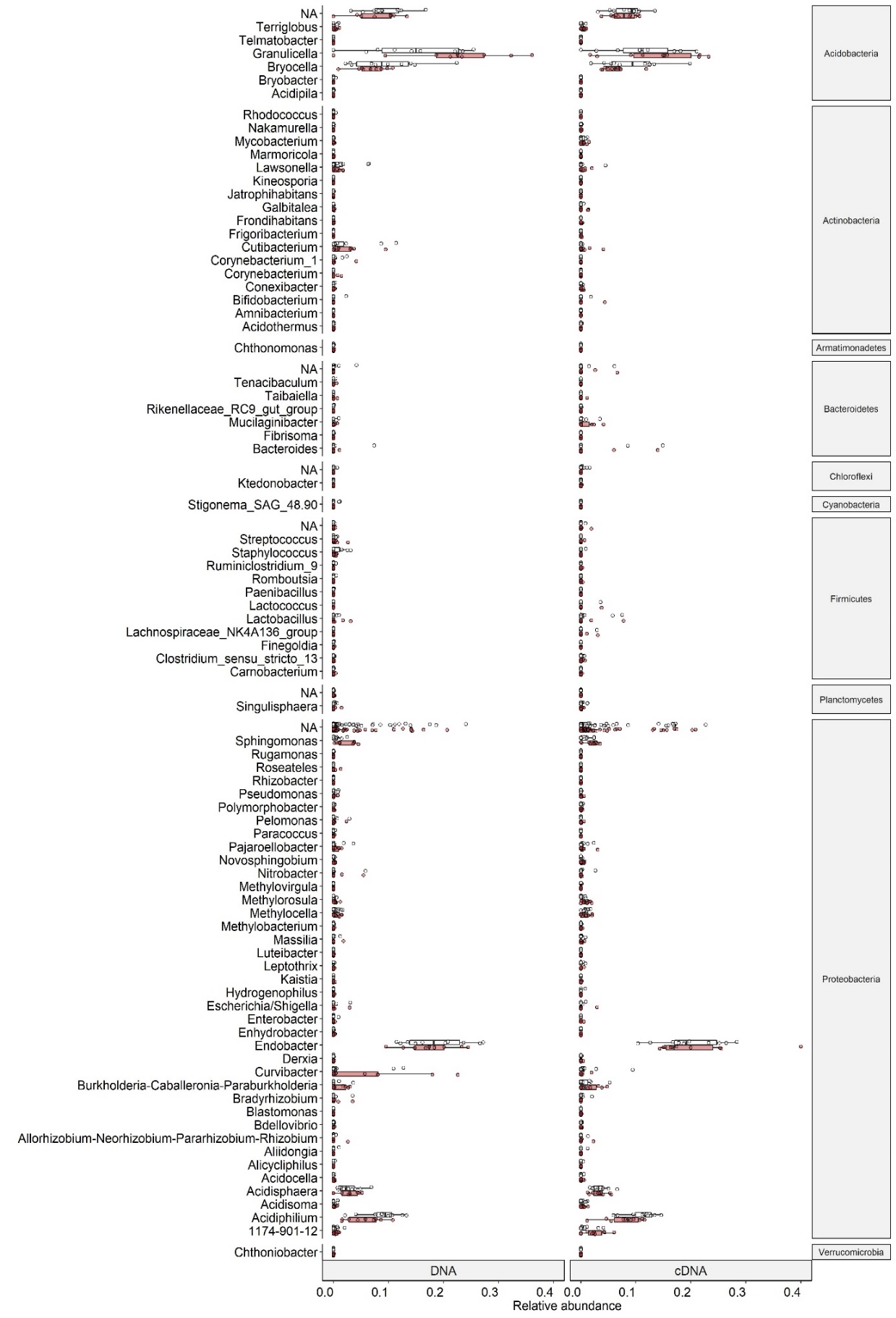


**Supplementary Figure 8** Fixed effect structure of the linear mixed-effect model testing the effect of treatment, *Betula nana* abundance and litter abundance on the 16S rRNA gene copy numbers. Non-overlapping 95% High Posterior Density Credible Interval (95% CrI) are used to detect significant differences between effects. Parameters with 50% CrI overlapping 0 are indicated by open circles. Parameters with 50% CrI not overlapping 0, but with 95% CrI overlapping 0 are indicated by closed black circles. Thick lines represent 50% CrI and thin lines represent 95% CrI.


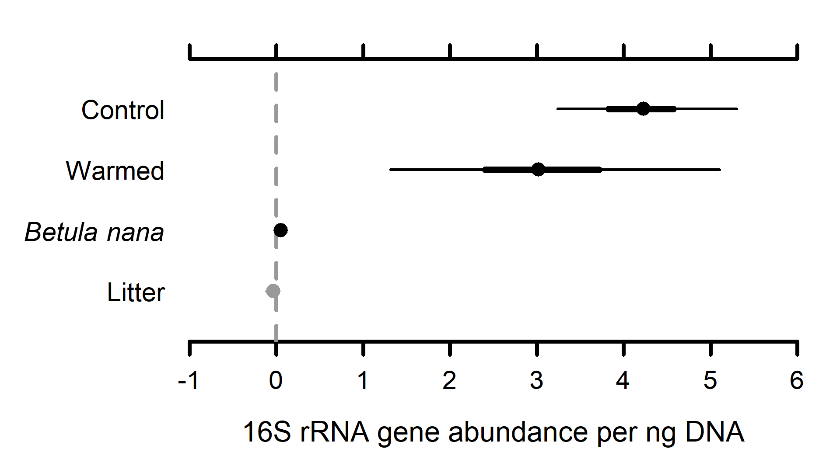


**Supplementary Figure 9** Fixed effect structure of the linear mixed-effect model testing the effect of treatment, *Betula nana* abundance and litter abundance on the *nifH* gene copy numbers. Non-overlapping 95% High Posterior Density Credible Interval (95% CrI) are used to detect significant differences between effects. Parameters with 50% CrI overlapping 0 are indicated by open circles. Parameters with 50% CrI not overlapping 0, but with 95% CrI overlapping 0 are indicated by closed black circles. Thick lines represent 50% CrI and thin lines represent 95% CrI.


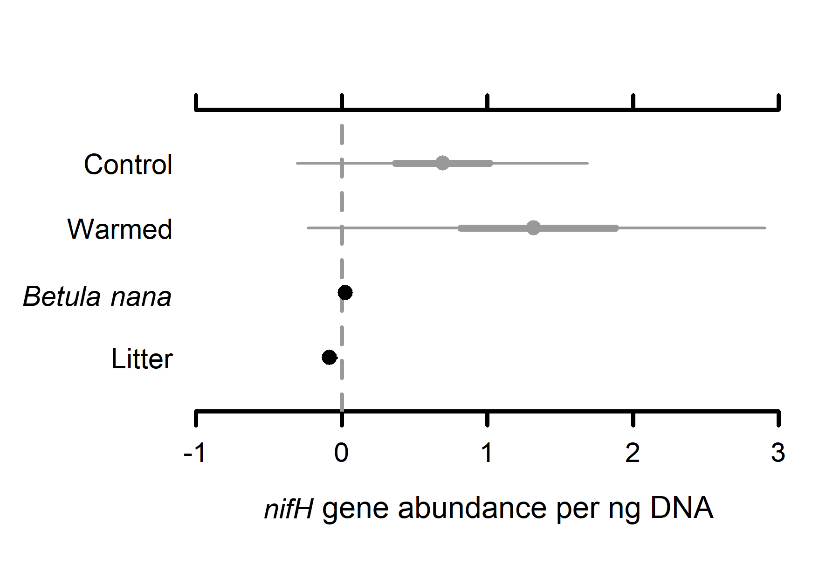


## Supplementary Tables

**Supplementary Table 1** Temperature and relative humidity for the OTC (warmed) and control plots measured in June-August 2016 (temperature and relative humidity 10 cm above the moss layer) and August 2018-June 2019 (temperature on the moss surface). Shown are mean ± standard error of the mean. Significant differences (t-test, *P* < 0.05) are indicated in bold.

| Air temperature  June – August 2016 | | | Moss surface temperature  August 2018 – June 2019 | | | Relative humidity Air  June – August 2016 | | |
| --- | --- | --- | --- | --- | --- | --- | --- | --- |
| OTC | Control | ∆°C | OTC | Control | ∆°C | OTC | Control | ∆% |
| 11.4  ± 0.1 | 10.0  ± 0.1 | **1.4** | 1.28  ± 0.01 | 1.06  ± 0.01 | **0.22** | 78.8  ± 0.36 | 81.8  ± 0.37 | **-3** |

**Supplementary Table 2** Summary for the Permanova testing the effect treatment, Betula nana abundance and litter abundance on the DNA-based bacterial community variation of the lichen.

|  | DF | Sum of Squares | Mean Squares | F.Model | R^2^ | Pr(>F) |
| --- | --- | --- | --- | --- | --- | --- |
| Treatment | 1 | 0.6646 | 0.66461 | 2.7591 | 0.07019 | <0.001 |
| *Betula nana* | 1 | 0.3654 | 0.36536 | 1.5168 | 0.03859 | 0.1646 |
| Litter | 1 | 0.2483 | 0.24830 | 1.0308 | 0.02622 | 0.6616 |
| Residuals | 34 | 8.1900 | 0.24088 |  | 0.86499 |  |
| Total | 37 | 9.4683 |  |  | 1.00000 |  |

**Supplementary Table 3** Summary for the Permanova testing the effect treatment, Betula nana abundance and litter abundance on the cDNA-based bacterial community variation of the lichen.

|  | DF | Sum of Squares | Mean Squares | F.Model | R^2^ | Pr(>F) |
| --- | --- | --- | --- | --- | --- | --- |
| Treatment | 1 | 0.6183 | 0.61835 | 3.1828 | 0.06659 | <0.001 |
| *Betula nana* | 1 | 0.5283 | 0.52833 | 2.7194 | 0.05690 | 0.06879 |
| Litter | 1 | 0.3674 | 0.36736 | 1.8909 | 0.03956 | 0.04850 |
| Residuals | 40 | 7.7712 | 0.19428 |  | 0.83694 |  |
| Total | 43 | 9.2852 |  |  | 1.00000 |  |
